# Association of Tinnitus with Depression in a Normal Hearing Population

**DOI:** 10.3390/medicina57020114

**Published:** 2021-01-27

**Authors:** Jay Choi, Chang Ho Lee, So Young Kim

**Affiliations:** Department of Otorhinolaryngology-Head and Neck Surgery, CHA University College of Medicine, 59, Yatap-ro, Bundang-gu, Seongnam 13496, Korea; jay.choi9102@gmail.com (J.C.); hearwell@gmail.com (C.H.L.)

**Keywords:** tinnitus, hearing, depression, risk factors

## Abstract

*Background and Objectives*: The relationship between depression in tinnitus patients without hearing loss remains elusive. This study aimed to investigate the association between tinnitus and normal hearing and depression. *Materials and Methods*: Participants aged ≥12 years with normal hearing levels were recruited from the Korea National Health and Nutrition Examination Survey (KNHANES), 2009–2012. Participants with normal hearing were divided into the tinnitus and non-tinnitus groups. The relationship between tinnitus with normal hearing and variables including age, sex, depression, ischemic heart diseases, stroke, diabetes, hypertension, dyslipidemia, chronic renal disease, noise exposure, and depression were analyzed. The odds of depression for tinnitus with normal hearing were estimated using multiple logistic regression tests with complex sampling. *Results*: The results showed that 4.9% (107/2221) and 2.8% (290/10,316) of participants in the tinnitus group and the non-tinnitus group, respectively, experienced depression (*p* < 0.001). Sex, ischemic heart disease, dyslipidemia, noise exposure, and depression were positively related to tinnitus with normal hearing. The odds ratio of depression for tinnitus with normal hearing were 1.89 (95% CI 1.37–2.60, *p* < 0.001). *Conclusions*: Tinnitus with normal hearing was related to the female sex, ischemic heart disease, dyslipidemia, noise exposure, and depression. Depression had the highest odds of tinnitus with normal hearing.

## 1. Introduction

Tinnitus is a symptom that impairs the quality of life. One study reported that the prevalence of tinnitus was as high as 34.4%, based on the characteristics of the study population [1]. In Korea, a study reported that approximately 20.7% of the adult population suffered from tinnitus [2]. The underlying pathophysiological mechanisms of tinnitus remain controversial. The elevated neural synchrony in auditory systems including the dorsal cochlear nucleus is thought to evoke the perception of tinnitus [3]. Therefore, tinnitus is largely related to hearing loss including noise-exposed hearing loss and presbycusis [4,5]. However, tinnitus can occur without any definite auditory deficits. Cochlear synaptopathy, which refers to problems in the connections between inner hair cells and auditory nerves, was suggested as one of the causes, whereas hidden hearing loss was suspected to be the cause of tinnitus in subjects with clinically normal hearing [6]. Apart from auditory problems, one study suggested that central disinhibition, irrespective of peripheral differentiation, could induce tinnitus in subjects with normal hearing [7].

Due to multiple pathophysiological factors, tinnitus has been reported to be associated with several comorbidities. Tinnitus is associated with an elevated risk of cerebrovascular diseases (adjusted odds ratio [AOR] = 1.66, 95% confidence intervals [95% CI] = 1.34–2.04) [8]. Furthermore, other comorbidities including dyslipidemia, osteoarthritis, rheumatoid arthritis, asthma, thyroid diseases, and depression have been reported to be related to tinnitus [2]. Common vascular and inflammatory pathophysiologic factors have been suggested for the occurrence of tinnitus and comorbidities [8]. However, the impact of hearing loss on tinnitus could have influenced these results because they did not consider the level of hearing loss. Therefore, the relationship between hearing loss and comorbidities could be mixed to determine the association between tinnitus and comorbidities in previous studies.

Clinically, a considerable number of young-to-middle-aged patients visit clinics with tinnitus without hearing loss in audiometric findings. Approximately 4.7–46% of children with normal hearing were estimated to have tinnitus [9,10]. The pathophysiology of tinnitus with normal hearing is still unclear, and potential etiologies have been proposed including the impairment of central auditory processing with some conflicts [11,12]. The current study postulated that tinnitus in the population with normal hearing might be less related to age and comorbidities, but related more to psychological or emotional problems such as depression. In addition, the prevalence of tinnitus in a population with normal hearing is not clearly known. To prove this hypothesis and to solve this problem, the current study explored the prevalence and factors associated with tinnitus in a population with normal hearing. To the best of our knowledge, no prior study has reported the prevalence of tinnitus in a population with normal hearing. In addition, the impact of depression on tinnitus was investigated based on the age and sex of the individuals.

## 2. Materials and Methods

### 2.1. Ethical Considerations

The Ethics Committee of Bundang CHA Medical Center approved the present study (IRB No. 2018-12-046, Approval date: 10 January 2019). Before the survey, the study was explained to all participants or legal representatives of minors, and written informed consent was obtained. The data source for the present study was the Korea National Health and Nutrition Examination Survey (KNHANES), which included participants who represented the South Korean population [13]. The survey included three main categories: health interviews, nutritional surveys, and physical examinations. Statistical methods were applied based on the sampling design and weighted values were utilized. The KNHANES data collected by the Centers for Disease Control and Prevention of Korea from 2009 to 2012 were analyzed. Each year, 192 districts were chosen by a panel, and 20 households in each of these districts were further identified to allow sampling that reflected the entire Korean population. Stratification of the sample was performed using a multi-stage, clustered sampling method, weighted, and adjusted by statisticians who performed post-stratification and accounted for the non-response rates and extreme values.

**Figure 1:**
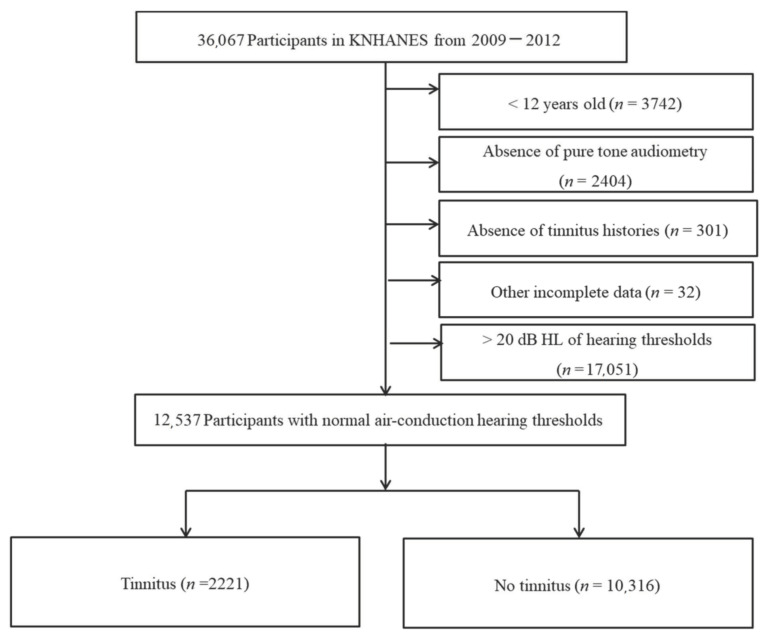
Schematic illustration of the participant selection process that was used in the present study. Of a total of 36,067 participants, 2221 tinnitus participants and 10,316 no tinnitus subjects were analyzed.

Among a total of 36,067 participants in the KNHANES from 2009 to 2012, participants with normal hearing were selected. Participants who did not undergo pure-tone audiometry tests were excluded. As pure tone audiometry tests were performed only in participants ≥12 years of age in the KNHANES, individuals who did not meet the criteria were excluded. In addition, participants with missing demographic data or past medical histories of diabetes, hypertension, dyslipidemia, ischemic heart disease, stroke, chronic renal disease, noise exposure, and depression were excluded. Ultimately, 12,537 participants were included in the present study (Figure 1).

### 2.2. Variables

The presence of tinnitus was surveyed by asking the question, “Have you heard a sound(s) (buzzing, hissing, ringing, humming, roaring, or machinery noise) in your ear in the past year?” [14]. Participants who answered “yes” were classified as the tinnitus group and those who answered “no” were classified as the non-tinnitus group. The presence of hearing loss was examined using the pure-tone air-conduction threshold test in a sound-proof booth and audiometer (GSI SA-203; Entomed Diagnostics AB, Lena Nodin, Sweden) [15]. Hearing loss was defined as >20 dB HL of the average air-conduction hearing threshold at 0.5, 1, 2, and 3 kHz [16].

Depression was diagnosed by a psychiatrist. The past medical histories of diabetes mellitus, hypertension, dyslipidemia, ischemic heart disease, stroke, and chronic renal diseases were classified based on diagnosis by physicians and reported by the participants. Noise exposure was surveyed by asking the question; “Have you ever been exposed to loud noise (causing a need to raise one’s voice during a conversation, such as while traveling in a car, truck, or motorcycle, machine sound, or concerts) for more than 5 h a week?” Participants who answered “yes” were classified as positive for noise exposure.

### 2.3. Statistical Analyses

Logistic regression tests with complex sampling were performed for explanatory variables including age, sex, depression, ischemic heart diseases, stroke, diabetes, hypertension, dyslipidemia, chronic renal disease, noise exposure, and depression to examine which factors will influence tinnitus. Two-tailed analyses were conducted, and the statistical analyses that yielded a *p*-value < 0.05 were considered statistically significant. Additionally, the samples were also adjusted for a combination of age, sex, diabetes mellitus, hypertension, dyslipidemia, ischemic heart disease, stroke, chronic renal disease, noise exposure, and depression to determine which of the characteristics had a stronger association with tinnitus. To determine whether age was attributed to the differences in the likelihood of depression in the tinnitus group, the current study divided the samples into three categories based on age: young, 12≤, and ≤29 years; middle, 30≤, and ≤59 years; and old, ≥60 years. Using the statistical method for the crude dataset, the AOR and 95% CI were calculated. The results were statistically analyzed using SPSS ver. 22.0 (IBM, Armonk, NY, USA).

## 3. Results

A total of 12,537 Korean participants were included in the study (Table 1). The mean age of participants with tinnitus was 38.58 years (standard deviation [SD] = 16.53) and of those without tinnitus was 39.09 years (SD = 15.80). Among those with tinnitus, the number of female participants was higher than that of males (females, 1410/2221, 63.5%; males, 5842/10,316, 56.5%). In contrast, 4.9% of the tinnitus group had depression, and 2.8% of the no-tinnitus group experienced depression (chi-squared test, *p* < 0.001).

Logistic regression analysis revealed that tinnitus had a positive association with sex, depression, ischemic heart disease, dyslipidemia, and noise exposure. Multiple logistic regression analysis also yielded similar results, showing that sex, ischemic heart disease, dyslipidemia, noise exposure, and depression were positively associated with tinnitus (Table 2). Females were more likely than males to have tinnitus (AOR = 1.75, 95% CI = 1.53–1.99, *p* < 0.001). There was an increase in the rates of dyslipidemia by 1.42-fold (95% CI = 1.11–1.82, *p* = 0.008), ischemic heart disease by 1.70-fold (95% CI = 1.05–2.76), and noise exposure was observed in participants with tinnitus. The adjusted odds of depression in tinnitus were highest among the analyzed variables (AOR = 1.89, 95% CI = 1.37–2.60).

In the full cohort, age was not associated with the probability of tinnitus (AOR = 0.99, 95% CI = 0.99–1.00, *p* = 0.004). However, more significant results were achieved by dividing the selected population into three age-based categories (Table 3). Only the middle-aged group (between 30 and 59 years of age) was positively associated with tinnitus and depression (AOR = 1.89, 95% CI = 1.37–2.60, *p* < 0.001). According to sex, both male and female subgroups demonstrated higher odds of depression in tinnitus (AOR = 2.88, 95% CI = 1.57–5.29, *p* = 0.001 in males and AOR = 1.66, 95% CI = 1.18–2.33, *p* = 0.004 in women).

## 4. Discussion

The prevalence of tinnitus was estimated to be 17.7% (2221/12,537) in a population with normal hearing. Approximately 4.9% of tinnitus patients with normal hearing suffered from depression. Tinnitus patients with normal hearing showed higher odds for sex, dyslipidemia, ischemic heart disease, and depression in the present study. They demonstrated 1.89-fold higher odds for depression than the no-tinnitus group (95% CI = 1.37–2.60). The association between tinnitus with normal hearing and depression was evident in both sexes and the middle age (30–59 years old) population (AOR = 1.89, 95% CI = 1.37–2.60).

Depression was related to tinnitus with normal hearing in the current study. Several previous studies have reported elevated rates of depression in patients with tinnitus [17,18]. A cross-sectional study using a national health survey demonstrated 4.8-fold higher odds of depression in patients with tinnitus (95% CI = 3.5–6.7, *p* < 0.001) [17]. However, the study did not consider the level of hearing of the participants; thus, the influence of hearing loss in tinnitus could not be distinguished from the relationship between tinnitus and depression. Another study demonstrated a positive correlation between tinnitus and symptoms of depression [18]. Specifically, the frequency of tinnitus correlated with depression (*r* = 0.6, *p* = 0.001), and the intensity correlated with the Beck Depression Scale (*r* = 0.28, *p* = 0.13) [18]. However, the study included a small population size (*n* = 44) without a control group. In addition, other potential confounding factors including age, sex, and past medical history were considered. Similar to the results of the present study, a previous study reported an association between depression and tinnitus in participants with normal hearing [19]. Approximately 41.7% (35/84) of tinnitus participants with normal hearing had depression, compared to 4.3% (2/47) of non-tinnitus participants with normal hearing (*p* < 0.001) [19]. However, the study had limitations of unconcerned confounders and a small study population, as in previous studies. The results of the present study showed higher odds of depression in tinnitus patients with normal hearing, even after considering other confounders such as age, sex, past medical history, and noise exposure. In addition, depression demonstrated the highest odds of tinnitus with normal hearing among the analyzed variables. According to age, only the middle-aged population showed an association between tinnitus and depression in this study. A cross-sectional study also demonstrated that there was no association between tinnitus and depression in children aged 6–16 years [20]. Aside from depression, other pathophysiologic contributors might have a role in younger patients with tinnitus [21].

Several molecular and imaging studies have supported the association between tinnitus and depression. The neural connections between the tinnitus-generating centers of the dorsal cochlear nucleus and non-auditory brainstem structures of the locus coeruleus, reticular formation, and raphe nuclei, which have roles in emotional response, could partially explain the association between tinnitus and depression [22]. In addition, the importance of serotonergic neural transmission was reported in a genetic study, which demonstrated that the polymorphism of the serotonin transporter gene (SLC6A4) was associated with the visual analog scale of tinnitus [23]. More broadly, various non-auditory brain areas such as the limbic system have been suggested to demonstrate abnormal neuronal activities in neuroimaging studies, therefore, it was proposed that thalamocortical dysrhythmia might result in tinnitus [24].

The common chronic diseases, hypertension and diabetes, were not associated with tinnitus with normal hearing in the present study. Tinnitus in the general population was reported to be related to various comorbidities including hypertension, diabetes, chronic renal disease, and stroke [25]. The lesser association between tinnitus and these chronic diseases could be attributed to the lower effect of auditory deprivation on tinnitus in the present study design in participants with normal hearing. For instance, the association between tinnitus and hypertension was presumed to be due to a compromise in cochlear microcirculation, which results in hearing loss [25]. Similarly, the association between hearing loss and other comorbidities such as diabetes, chronic renal disease, and stroke has been described. Moreover, the younger study population in the current study had a lower frequency of comorbidities compared to other studies that included participants with hearing loss, which might have attenuated the effects of these comorbidities. Thus, the present results on the association between tinnitus and depression improved the previous findings by excluding the confounding effects of tinnitus-related comorbidities and hearing loss.

As average hearing thresholds were calculated using frequencies of 0.5, 1, 2, and 3 kHz, the potential impact of high- or low-frequency hearing loss could not be excluded [26]. In addition, hidden hearing loss with abnormal brainstem response latency or outer hair cell dysfunction, which could be evaluated using distortion-product optoacoustic emissions or auditory brainstem response, was not considered in this study. It was reported that personal music player use and noise exposure were related to the experience of tinnitus in children, although it did not frequently accompany hearing loss [27]. Central deafferentation due to cochlear damage, which was measured by a threshold equalizing noise test and a pitch scaling task, existed even in the tinnitus patients with normal audiograms of 0.25, 0.5, 1, 2, 3, 4, 5, 6, and 8 kHz [28]. Thus, the effects of deafferentation or cochlear damage in patients with tinnitus could not be excluded based on pure tone audiometry, even with lower or higher frequency thresholds. The present results need to be interpreted as the effects of tinnitus on depression and other comorbidities in the minimized condition of hearing disturbance, which affects communication and social activities. The hearing loss, in the absence of tinnitus, was reported to influence depression and comorbidities.

The current study was based on a large, representative study population, which improved the statistical power and fidelity of the current results. In addition, the presence of hearing loss was examined using pure tone audiometry, thereby enhancing the accuracy of the data. However, there were some limitations to be considered. As the KNHANES is a cross-sectional survey and did not survey the onset of tinnitus or depression, the temporal association between depression and tinnitus could not be delineated in the present study. Tinnitus was self-reported based on the survey item and tinnitograms were lacking in this study. The duration and types of tinnitus were not differentiated in this study. Objective tinnitus related to vascular abnormalities, palatal myoclonus, stapedial/tensor tympani muscle spasm or somatosensory tinnitus, and temporomandibular joint diseases could occur in subjects with normal hearing [29,30]. The history of antidepressant therapy was not considered in this study, which could have influenced the association between tinnitus and depression. In addition to the possible inclusion of patients with high or low-frequency hearing loss, the laterality, severity, and pitch of tinnitus varied among participants in the tinnitus group. A recent study reported the difference in audiological profiles between patients with bilateral and unilateral tinnitus [31], which could help delineate the underlying pathophysiology of tinnitus.

## 5. Conclusions

A total of 17.7% of the population with normal hearing had tinnitus. Depression demonstrated the highest positive association with tinnitus in subjects with normal hearing including age, sex, and past medical histories of hypertension, diabetes, dyslipidemia, ischemic heart disease, stroke, chronic renal disease, and noise exposure.

## Figures and Tables

**Table 1:** General characteristics of participants.

Characteristics	Total Participants	
	Tinnitus (*n*, %)	No Tinnitus (*n*, %)
Number	2221	10,316
Age (years old)	38.58 (16.53)	39.09 (15.80)
Sex		
Male	811 (36.5)	5842 (56.6)
Female	1410 (63.5)	4474 (43.4)
Diabetes mellitus		
Yes	87 (4.0)	399 (3.9)
No	2113 (96.0)	9831 (96.1)
Hypertension		
Yes	240 (10.9)	1158 (11.3)
No	1960 (89.1)	9072 (88.7)
Dyslipidemia		
Yes	177 (8.0)	700 (6.8)
No	2023 (92.0)	9530 (93.2)
Ischemic heart diseases		
Yes	40 (1.9)	94 (1.0)
No	2050 (98.1)	9567 (99.0)
Stroke		
Yes	14 (0.6)	60 (0.6)
No	2186 (99.4)	10,170 (99.4)
Chronic renal diseases		
Yes	6 (0.3%)	21 (0.2%)
No	2194 (99.7)	10,209 (99.8)
Depression		
Yes	107 (4.9)	290 (2.8)
No	2093 (95.1)	9940 (97.2)

**Table 2:** Crude and adjusted odds ratios (95% confidence interval) of each variable for tinnitus.

Characteristics	Tinnitus			
	Crude	*p*-Value	Adjusted ^†^	*p*-Value
Age	1.00 (0.99–1.00)	0.028	0.99 (0.99–1.00)	0.004
Sex	1.49 (1.32–1.67)	<0.001 *	1.75 (1.53–1.99)	<0.001 *
Diabetes	1.01(0.75–1.37)	0.929	1.07 (0.77–1.48)	0.694
Hypertension	0.91 (0.75–1.10)	0.313	0.92 (0.74–1.15)	0.246
Dyslipidemia	1.28 (1.03–1.58)	0.025 *	1.42 (1.11–1.82)	0.008 *
Ischemic heart diseases	1.60 (1.03–2.49)	0.035	1.70 (1.05–2.76)	0.030
Stroke	1.27 (0.63–2.58)	0.507	1.31 (0.62–2.80)	0.479
Chronic renal diseases	0.86 (0.29–2.55)	0.785	0.70 (0.21–2.32)	0.560
Depression	1.97 (1.45–2.67)	<0.001 *	1.89 (1.37–2.60)	<0.001 *

* Cox-proportional hazard regression model, Significance at *p* < 0.05. ^†^ Adjusted model for age, sex, diabetes mellitus, hypertension, dyslipidemia, ischemic heart disease, stroke, chronic renal diseases, and depression.

**Table 3:** Crude and adjusted odds ratios (95% confidence interval) of depression for tinnitus.

Characteristics	Tinnitus			
	Crude	*p*-Value	Adjusted ^†^	*p*-Value
Male	2.93(1.59–5.40)	0.001 *	2.88 (1.57–5.29)	0.001 *
Female	1.52 (1.09–2.11)	0.014 *	1.66 (1.18–2.33)	0.004 *
Young (12–29 years)	1.67 (0.85–3.25)	0.134	1.77 (0.88–3.57)	0.111
Middle (30–59 years)	1.97 (1.45–2.67)	<0.001 *	1.89 (1.37–2.60)	<0.001 *
Old (≥60 years)	1.26 (0.60–2.62)	0.541	1.28 (0.58–2.80)	0.545

* Significance at *p* < 0.05. ^†^ Adjusted model for age, sex, diabetes mellitus, hypertension, dyslipidemia, ischemic heart disease, stroke, chronic renal diseases, and noise exposure.

## Data Availability

Releasing of the data by the researcher is not allowed legally. All of data are available from the database of the Korea National Health and Nutrition Examination Survey (KNHANES). KNHANES allows all of this data for the any researcher who promises to follow the research ethics with some cost. If you want to access the data of this article, you could download it from the website after promising to follow the research ethics.
